# In which rounds were the most rotations of key players made, and how did this affect physical activity? Analysis of the eight best teams of the 2018 FIFA world cup Russia

**DOI:** 10.1186/s13102-024-00834-4

**Published:** 2024-02-15

**Authors:** Paweł Chmura, Michał Kołodziejczyk, Marcin Andrzejewski, Jan Chmura, Andrzej Rokita, Adrian Drożdżowski, Krzysztof Maćkała, Marek Konefał

**Affiliations:** 1 Department of Team Games, Wroclaw University of Health and Sport Sciences , I.J. Paderewskiego 35, 51612 Wrocław, Poland; 2 Department of Methodology of Recreation, Poznań University of Physical Education, Królowej Jadwigi 27/39, 61871 Poznań, Poland; 3 Department of Human Motor Skills, Wroclaw University of Health and Sport Sciences , I.J. Paderewskiego 35, 51612 Wrocław, Poland; 4 Department of Track and Field, Wroclaw University of Health and Sport Sciences , I.J. Paderewskiego 35, 51612 Wrocław, Poland

**Keywords:** Soccer, Key players, Match analysis, Match performance, Squad rotation, Starting line-up

## Abstract

**Background:**

Team management, especially player selection, rotation, and availability, are critical issues when dealing with the high demands of modern training and gameplay. As such, research continuously seeks ways to improve these actions or implement new ideas to gain a competitive advantage through the rotation of players in the starting line-up. The current study aimed to examine the rounds of the 2018 FIFA World Cup Russia in which the most rotations of key players were made and how this affected physical activity.

**Methods:**

The sample consisted of 110 players from the top eight teams in the 2018 World Cup Russia who played entire matches for up to 90 min in seven consecutive games. All players were divided into key players (KPs, *n* = 58) and non-key players (NKPs, *n* = 52). The analysis used data collected by an advanced motion analysis system known as STATS^®^, with physical activity variables analyzed, including total distance covered (TDC), distance covered with high intensity over 20 km/h (HIR), and the number of sprints undertaken. In statistical analysis, differences between categories and consecutive matches were calculated using the Kruskal-Wallis H test, and if a significant effect size was found, a multiple comparisons *p* values test was performed.

**Results:**

The best teams at the 2018 FIFA World Cup Russia used the most KP rotations with NKPs in the third match of the group stage. In addition, this was even more visible among more successful teams than less successful teams. The rotation strategy among the best eight teams allowed them to maintain the physical activity of KPs and NKPs in all rounds of the tournament.

**Conclusions:**

Coaches and coaching staff should incorporate squad rotation that includes a large group of players in their team management to improve their success. Team management expertise in player rotation during matches played over congested schedules at top tournaments maintains high levels of physical activity indicators (TDC, HIR, and sprints).

## Introduction

During the FIFA World Cup tournaments, teams must possess the highest possible level of physical activity in every match to fight to score the most goals. Maintaining such levels uses various regeneration strategies, among which is player rotation [1, 2]. Many studies indicate the positive aspects of using player rotation during the World Cup, where matches are played every four to five days [3–6]. A soccer team usually consists of key players (KPs), who appear in most matches throughout the season, and non-key players (NKPs), whose role is to replace KPs. Team rotation allows KPs a longer recovery, prevents overloads and injuries, creates a competitive environment within the team [7, 8], and allows NKPs to achieve higher activity, especially high-intensity running [9–11]. Smpokos-Sbokos et al. [12] confirmed the appropriateness of using rotation by showing that a low level of rotation can adversely affect players by, among other things, increasing dynamic stress load and residual fatigue. This raises the question of when and how often coaches make player rotations in tournaments and matches at the highest level of the sport.

Coaches often change line-ups between matches during domestic leagues, and only a small number of players play consistently in consecutive matches [13]. According to Carling et al. [13], squad rotation in soccer at the club level means that only 40% of KPs will have to play full matches in subsequent phases of the season. However, during tournaments such as the World Cup, coaches may limit the KP rotation due to the importance of each match. Therefore, despite the rotation, some players will play in most or all matches. Varley et al. [14] noted the use of rotation after a team qualified for the knockout stage after winning its first two matches in the group stage. In this situation, the coaches, confident of advancing to the knockout phase of the tournament, allowed KPs to rest. Varley et al. [14] also found that an average of three players per team played in all group-stage matches. However, when a team advances to the knockout stage, each subsequent match decides whether they will progress or be eliminated from the tournament. As such, it can be assumed that teams that reach the final rounds and succeed in the tournament adopt an optimal rotation strategy.

From a cognitive point of view, it is critical to understand how the player rotation strategy affects the physical activity of the team, with research showing that squad rotation during congested schedules contributed to improving or maintaining physical activity [5, 7, 15–17]. The KPs who play over 60 matches during the season, including league, cup, and national team matches, are the most exposed to overload. Indeed, a greater frequency of matches every four to five days increases the perceived load on muscle structures and the level of stress on players [18]. When playing two matches per week, an athlete’s ability to sprint, jump, and perform repetitive high-intensity exercises also decreases [19]. However, recent research indicates that the proper rotation of players by the coach can mask the signs of fatigue in congested schedules during tournaments such as the World Cup [20].

New strategies are constantly being sought to gain an advantage over the opponent, with team management through player rotation one of the key ways to deal with the high demands of modern football [21]. Currently, only one study by Kołodziejczyk et al. [22] described team management during the World Cup, but it only concerned the most exploited players among the top four teams. In the literature, there are only a few studies analyzing player rotation. Yet, it is crucial from the cognitive point of view to understand the rotation scheme/pattern used by the less successful and more successful teams, as well as the level of physical activity of the KPs and NKPs in the subsequent rounds of the tournament. Such knowledge seems attractive to physical preparation coaches and football coaches. Therefore, the purpose of this study was to examine in which rounds of the 2018 FIFA World Cup Russia the most rotations of KPs were made and how they affected physical activity.

## Material & methods

### Experimental approach to the problem

The study analyzed the top eight teams that reached at least the 2018 FIFA World Cup Russia quarter-finals. All players who played at least one full match were divided into KP and NKP groups, and the number of KP exchanges for NKPs in subsequent matches of the tournament was analyzed. These changes were also divided by the teams that made it to the quarter-finals (less successful - LS) and semi-finals (more successful - MS). In addition, we examined what level of physical activity in the subsequent matches of the tournament was characterized by KPs and NKPs.

An objective analysis of physical activity performed by professional soccer players employed the STATS® advanced motion analysis system (Stats LLC, IL, USA). An assumption was made that in the round with the most KP rotations, the NKPs replacing them would achieve higher physical activity parameters during the match, which may allow KPs to reach higher physical activity in the next round.

### Procedures

The research sample included the top eight teams (France, Croatia, Belgium, England, Russia, Sweden, Brazil, and Uruguay) over 38 matches of the 2018 FIFA World Cup Russia, with 110 players analyzed across 365 match observations (including 58 key players − 261 observations, and 52 non-key players − 104 observations). Due to the specificity of the position, goalkeepers were excluded from the analysis. The results obtained up to 90 min (without extra time) were used for the analysis. The mean height of the players was 183.28 ± 6.69 cm, body weight was 78.43 ± 7.68 kg, and age was 26.31 ± 3.44 years. The authors adopted two operational definitions of players for this study. KPs were all players who started the first match of the tournament and played until full-time. The NKPs were all other players who played in at least one full match in the tournament. The study maintained player anonymity in line with data protection laws and was conducted in accordance with the Declaration of Helsinki. In addition, the study design was approved by the local Ethics Board (No. 12/2021).

Physical activity measurement used an advanced motion analysis system known as STATS® (Stats LLC, IL, USA), operating at 25 frames per second to simultaneously track players every second of the game across all sections of the soccer field. Using cameras, the STATS system converts player movement on the field into quantitative data and generates reports. The validity and reliability of this system for making such measurements have been described in detail elsewhere [23, 24]. Match data was taken from the official FIFA website [25]. Data from the same resource was previously used in research related to FIFA World Cup analysis by da Mota et al. [26] and Kołodziejczyk et al. [22], who discussed the process of tracking and encoding the official FIFA dataset in detail.

The recorded variables included total distance covered (TDC), distance covered with high intensity over 20 km/h (HIR), and the number of sprints performed [10, 27–29].

### Statistical analysis

The Shapiro-Wilk and Levene tests were used. Arithmetic means, median, standard deviations, and quartile deviation were calculated (the description of physical activity presents arithmetic means ± standard deviation to allow for comparison with other studies). Differences between categories over consecutive matches were calculated using the Kruskal–Wallis H test. When a significant effect size was found, a multiple comparisons *p* values test was performed [30, 31]. The level of statistical significance was set at *p* < 0.05. All statistical analyses employed Statistica ver. 13.2 software package (StatSoft. Inc., CA, USA).

## Results

The statistical analysis of the total number of player rotations in relation to the subsequent rounds of the tournament revealed an effect for the total number of player rotations (H = 131.248(5); *p* = 0.001), the number of player rotations for MS teams (H = 118.286(5); *p* = 0.001), and the number of player rotations for LS teams (H = 57.170(3); *p* = 0.001), see Table 1.

**Table 1:** The number of player rotations in the starting line-up per team (median ± *Q*)

Variables	Type of player	Group stage				Knockout stage					H (sig)	MC (*p* ≤ 0.05)
		1st (1)	2nd (2)	3rd (3)	Round of 16 (4)		Quarter-final (5)	Semi-final (6)	Final/3rd place match (7)			
Number of rotation KPs on NKPs	Total	All KP	2.00 ± 0.50	5.00 ± 2.00	1.00 ± 1.50		2.00 ± 0.50	0.00 ± 0.00		1.00 ± 0.50	131.248 (0.001)	3 > 2,4,5,6,7; 6 < 2,3,4,5,7;
	More successful	All KP	1.00 ± 0.50	7.00 ± 1.00	0.00 ± 0.50		1.00 ± 1.00	0.00 ± 0.00		1.00 ± 0.50	118.286 (0.001)	3 > 2,4,5,6,7; 2,7 > 4,6;
	Less successful	All KP	2.00 ± 0.50	3.00 ± 0.25	3.00 ± 0.00		2.50 ± 0.50	-		-	57.170 (0.001)	2 < 3,4,5

KPs - Key players

NKPs– Non-key players

H - Kruskal–Wallis H test

(sig) - significance

MC - multiple comparisons *p* values test

𝑄– quartile deviation

The statistical analysis of the physical activity parameters in relation to the subsequent rounds of the tournament revealed no significant effect on the distance covered by KPs (H = 4.932(6); *p* = 0.553), the distance covered by NKPs (H = 2.787(5); *p* = 0.733); the HIR covered by KPs (H = 7.687(6); *p* = 0.262), the HIR covered by NKPs (H = 7.736(5); *p* = 0.171); the number of KP sprints (H = 9.285(6); *p* = 0.158), and the number of NKP sprints (H = 5.658(5); *p* = 0.341), see Table 2.

**Table 2:** Physical activity parameters up to 90 min of the best eight teams (means ± standard deviation)

Selected variables of physical activity	Key players/ Non-key players	Group stage			Knockout stage					H (sig)	MC (***p*** ≤ 0.05)
		1st (1)	2nd (2)	3rd (3)		Round of 16 (4)	Quarter-final (5)	Semi-final (6)	Final/3rd place match (7)		
Distance covered [km]	Key	9.87 ± 0.96	9.78 ± 0.96	9.35 ± 0.73		9.61 ± 1.11	9.82 ± 1.05	9.83 ± 0.99	9.79 ± 0.97	4.932 (0.553)	-
	Non-key	-	10.22 ± 1.16	10.02 ± 0.88		9.93 ± 0.94	10.14 ± 0.95	10.46 ± 1.13	10.47 ± 1.01	2.787 (0.733)	-
HIR [m]	Key	737.66 ± 297.16	625.24 ± 237.46	623.20 ± 261.78		655.70 ± 273.02	668.33 ± 260.43	670.11 ± 244.05	802.23 ± 315.62	7.687 (0.262)	-
	Non-key	-	752.13 ± 242.00	730.05 ± 264.22		654.89 ± 233.92	690.44 ± 191.02	907.57 ± 227.49	854.29 ± 249.42	7.736 (0.171)	-
Sprints [number]	Key	31.67 ± 11.85	27.48 ± 11.18	25.65 ± 10.73		28.05 ± 10.77	30.44 ± 12.08	29.25 ± 11.21	33.18 ± 10.68	9.285 (0.158)	-
	Non-key	-	31.40 ± 9.84	31.39 ± 11.18		29.79 ± 10.14	30.89 ± 7.92	39.57 ± 8.58	33.29 ± 9.67	5.658 (0.341)	-

H - Kruskal–Wallis H test

(sig) - significance

MC - multiple comparisons *p* values test

## Discussion

This study was designed to investigate the rounds in which the top teams of the 2018 FIFA World Cup Russia performed the most player rotations. Our main finding was that the greatest number of KP rotations for NKPs was used by the coaches in the third match of the group stage. Varley et al. [14] confirmed this by showing that player rotation may result from securing progression from the group stage after the first two matches. In addition, the high number of player rotations in the third round was even more pronounced among MS teams compared to LS teams. The four best teams of the tournament (MS teams) scored six points in the first two matches of the group stage and rotated almost all KPs in the third match (France - five rotations, Croatia - six, Belgium– seven, and England - eight). Research by Kołodziejczyk et al. [6] also investigated squad rotation at the 2018 FIFA World Cup Russia, with analysis of the top four teams of the tournament showing that coaches can manage the squad and potentially gain an advantage over their opponent by intentionally rotating the KPs at the right moments of the tournament [6]. Such a game strategy does not preclude potential promotion to the next phase of the competition, and coaches can use players who have not played before [14]. For comparison, the situation of the top four teams at the 2014 FIFA World Cup Brazil was evaluated, and it turned out that, despite winning the first two matches, only two of the four teams decided to increase the number of rotations during the third match of the group stage (data unpublished). It should also be taken into account that the situation of the teams and their game plan during the tournament could have been different had they not won the first two matches of the group stage. In our research, the number of KP rotations in subsequent rounds of the tournament was between two and three in LS teams. However, it is not easy to determine the precise model of team rotation during high-level competitions. Therefore, further analysis and observation of player rotation in world-class teams is encouraged.

Carling et al. [3] noted that squad rotation contributes to more effective KP recovery and leads to greater availability of these players. Thanks to this, coaches have greater comfort and the ability to select a larger group of players. According to Hillis et al. [32], another factor affecting the rotation of players may be an attempt to minimize the accumulation of fatigue before the knockout phase. Therefore, our next goal was to investigate how rotations affect the physical activity of KPs and NKPs. Our analysis showed that the increased squad rotation in the third round of the tournament affected the physical activity parameters of the team during the match. The regular introduction of NKPs contributed to more effective recovery and maintenance of physical activity for both KPs and NKPs in all rounds of the tournament. Such squad rotation extends the time for player regeneration, preventing a decrease in physical activity levels, especially in the knockout phase of the tournament. Indeed, Kołodziejczyk et al. [22] stated that it was very important for the teams because in the knockout phase, England played two extra-times, and the Croatians had three extra-times (in three consecutive matches).

Padrón Cabo et al. [33] demonstrated that NKPs are more rested and achieve better running parameters during the match in congested schedules. Our results confirmed these observations and showed that NKPs who replaced KP in the third match of the group stage achieved higher parameters in terms of TDC and HIR and generated more sprints than KPs at every stage of the tournament. Notably, the greatest TDC differences in favor of NKPs were recorded in the third match of the group stage and the sixth and seventh rounds of the tournament, from 0.63 to 0.68 km. In turn, the biggest difference in HIR and the number of sprints occurred in the semi-final, with 237 m and ten sprints, respectively. As noted earlier at the 2014 FIFA World Cup Brazil, the highest TDC, the HIR, and the total number of sprints were also achieved by the players in the semi-final match [34]. These findings show the high speed and endurance requirements of the players in the key rounds of soccer games at the highest level.

The physical activity of soccer players is characterized by high inter-match variability [35, 36]. From a statistical point of view, some differences may be insignificant due to high coefficients of variation. But from the point of view of practice, the longer distance run by the player during the match (more than 630 m, HIR of 237 m, and ten more sprints) is very significant for the effectiveness of the game [37]. This information is important from a practical point of view because the research conducted by Konefał et al. [38] showed that increasing the number of sprints performed significantly increased the chances of winning a match. Moreover, the importance of the ability of players to generate high intensity has been studied many times before by authors such as Dellal et al. [15] and Faude et al. [39] and shows a correlation with positive match results.

The authors are fully aware of many factors that may have influenced the analyses presented here. As this is a single tournament analysis, the data should be used with caution. Another limitation of this study is that technical and tactical activities were not included. Future analysis should also include physiological-biochemical variables and an assessment of perceived exertion (RPE). In addition, Woods et al. [40] showed another factor that can influence the decision of coaches to rotate the starting line-up, namely motivation. Therefore, it is worth examining this factor in future research also. Undertaking further research on player rotation strategies in top-level tournaments is important as such knowledge is essential, especially in tournaments where any loss can put the team at risk of being eliminated.

## Conclusions

The eight best teams at the 2018 FIFA World Cup Russia used the most KP rotations with NKPs in the third match of the group stage, which was even more evident among the MS teams compared to the LS teams. As noted, increased rotation affected the team’s physical activity parameters (TDC, HIR, and sprints) during the match, extended the time for player regeneration, and prevented a decrease in the level of physical activity, especially in the knockout phase of the tournament, as indicated by maintaining the physical activity of both KPs and NKPs in all rounds of the tournament. Moreover, NKPs achieved higher physical activity parameters throughout all phases of the tournament relative to the KPs. In conclusion, using increased squad rotation should be considered a strategy for KP regeneration and a method of maintaining player performance in aspects of the team’s physical activity in the crucial phases of the tournament.

### Practical application

Coaches and coaching staff should be aware of the opportunities offered by player rotation. Analysis of the best teams of the 2018 FIFA World Cup Russia shows that squad rotation is one of the key factors in increasing the chances of success. This recovery strategy will become increasingly important in future World Cups and congested schedules to counteract the deterioration of players’ performance. In addition, squad rotation can be the basis for optimizing the level of physical activity of KPs and NKPs by creating intra-team competition.

## Data Availability

Match data were retrieved from the official website of FIFA. The datasets used and/or analyzed in the study are available from the corresponding author upon reasonable request.
